# Seasonal dynamics and spatial distribution pattern of *Parapoynx crisonalis* (Lepidoptera: Crambidae) on water chestnuts

**DOI:** 10.1371/journal.pone.0184149

**Published:** 2017-09-01

**Authors:** Ni Li, Qi Chen, Jie Zhu, Xing Wang, Jian-Bin Huang, Guo-Hua Huang

**Affiliations:** 1 Hunan Provincial Key Laboratory for Biology and Control of Plant Diseases and Insect Pests, Changsha, China; 2 College of Plant Protection, Hunan Agricultural University, Changsha, China; Chinese Academy of Agricultural Sciences Institute of Plant Protection, CHINA

## Abstract

*Parapoynx crisonalis* (Walker, 1859) (Lepidoptera: Crambidae) is a major pest of aquatic vegetables and aquatic landscape plants. It has been responsible for causing considerable economic damage to water chestnut (*Trapa natans*) plants. In the Changsha vicinity of China, *P*. *crisonalis* has five generations a year. Populations of *P*. *crisonalis* were relatively low in April and began to rapidly rise at the beginning of May. At the end of July and early August, the population dropped dramatically. A rebound occurred at the end of August and early September, which was referred to as the second population peak. From then, until early November, the *P*. *crisonalis* population steadily diminished in preparation for overwintering. The primary factors influencing the seasonal dynamics of *P*. *crisonalis* were the climatic conditions, especially the temperature, and secondarily precipitation. Between May and October, the *P*. *crisonalis* adults were evenly distributed in the pond. In May and June, the eggs of *P*. *crisonalis* were present in an aggregate distribution, due to the effects of environmental heterogeneity. In July and August, however, they were found to be in a uniform distribution.

## Introduction

*Parapoynx crisonalis* (Walker, 1859) (Lepidoptera: Crambidae: Nymphulinae) is a widespread pest that is widely distributed throughout Asia including China (Jiangsu, Zhejiang, Anhui, Jiangxi, Fujian, Taiwan, Hubei, Hunan, Guangdong, Sichuan, Guizhou, and Guangxi Provinces), Japan, Indonesia, Burma, Thailand, Sri Lanka, India, and in Australia [1–3]. It has also recently become established in the British Isles [4]. *P*. *crisonalis* feeds on numerous wild aquatic plants as well as cultivated ornamental species, and many aquatic vegetables, such as: *Trapa natans* L., *Nymphoides peltatum* (Gmel.) O.Kuntze, *Euryale ferox* Salisb, *Nymphaea tetragona* Georgi, etc. [5, 6]. Damage to the host plant is primarily through skeletonizing of the leaves caused by *P*. *crisonalis* larval feeding [7, 8]. *P*. *crisonalis* are tolerant of a wide temperature range for their growth and development, and are able to successfully complete their life cycle between 21°C and 36°C, with the optimal development temperature being between 24°C ~ 30°C [9]. The extent of damage caused by *P*. *crisonalis* has been recognized and its importance as an aquatic pest of vegetables in China documented [3].

In order to develop a comprehensive ecological pest management plan, it is crucial to completely understand the ecology of the pest. Elucidating the seasonal dynamics of a species is a core scientific issue in the study of insect population ecology. Seasonal dynamics is more inclusive than just the normal growth and decline of a population; it involves the number and distribution of biological species that are constantly changing over time while also showing seasonal fluctuations and interannual variability. Insect populations normally increase and decrease around a mean density, with the normal trend, which returns to the original level, being called dynamic equilibrium. The study of seasonal dynamics mainly includes quantity, structure, regularities of distribution, spatial dynamic changes, influencing factors and mechanisms. It can quantitatively describe the population change rule and relationship among different influencing factors. These factors are divided into two aspects consisting of biotic and abiotic factors [10]. The biotic factors mainly include conspecific individuals, food sources, and natural enemies. The abiotic factors, also called physical factors, include temperature, precipitation, wind, humidity, light and pesticides. Among the abiotic factors, temperature stands out as one of the most critical factors in insect seasonal dynamics [11, 12]. We combined the available information regarding seasonal dynamics with our field survey data to analyze the annual and seasonal fluctuations of *P*. *crisonalis*.

Insect population spatial pattern is another major topic in the study of insect ecology and pest control systems. Research on spatial patterns also helps to understand the interaction that may occur between random population change processes and the insect population as a whole. The aggregation degree of a population spatial pattern can describe some characteristics of population ecology and regulation mechanisms involving density, such as foraging and reproduction under certain circumstances, territorial behavior, diffusion behavior, communication behavior, etc. Codling moth (*Cydia pomonella* L.) larvae, after locating a pupation site, attract conspecific larvae staying together by an aggregation pheromone produced by the cocooning larvae [13]. Aggregation behavior was found to contribute to foraging among an insect population by Sánchez *et al*., 2009 [14]. Citrus leafminers were found to prefer the terminal leaves of younger shoots, where eggs were usually concentrated in oviposition sites [15].

The objective of this study was to assess the annual and seasonal regularity and spatial pattern of *P*. *crisonalis* population in the field. We report the annual life history, the effects of temperature and precipitation on populations of *P*. *crisonalis*, the numerical trend of each stage of *P*. *crisonalis* on *T*. *natans*, and the degree of aggregation occurring each month.

## Materials and methods

### Experimental protocol

Field experiments on the seasonal dynamics of *P*. *crisonalis* were conducted using water chestnut plants as hosts in two ponds located on the Hunan Agricultural University, Changsha, Hunan Province (N28°10′47.28″, E113°04′48.89″). The water chestnut leaves open on the surface of the water beginning in mid-April in the Changsha area. The five-spot-sampling method was used to select five field samples. Single sample was 1m x 1m. We tracked the total numbers of *P*. *crisonalis* and recorded the number of individuals in each instar during every generation for each field sample. During the early growing season when population densities were at low level, we used a combined random sampling method to determine the field sample in areas where *P*. *crisonalis* were present. Observations were conducted every other day until the last of the water chestnut plants had died. The survey was begun in April 2014 and ended in May 2015.

The *P*. *crisonalis* population gradually increased during April and reached the population peak period during October in 2014. The five-spot-sampling method was used to determine the spatial distribution pattern of *P*. *crisonalis*. Twenty strains of water chestnut in each sample point were randomly selected and the numbers of *P*. *crisonalis* eggs, larvae, pupae and adults found on the leaves of each strain were recorded every other day. This survey was also conducted from April 2014 to May 2015.

The meteorological data was provided by the Meteorological Data Center of China, Meteorological Administration (CMA, http://data.cma.cn/), the National Centers for Environmental Prediction (NCEP, http://www.ncep.noaa.gov/), and the National Center for Atmospheric Research (NCAR, http://ncar.ucar.edu/).

### Spatial distribution pattern

The data during the population peak period was analyzed using the following indices, which were used to determine the type of spatial distribution pattern.

(1) Diffusion coefficient (*C*) [16];

$$C=S^{2}/m$$

Where *S*^2^ is the variance, *m* is the mean density of *P*. *crisonalis* on each water chestnut plant. The spatial distribution pattern is aggregative, random and uniform when *C*>1, *C* = 1 and *C*<1, respectively.

(2) The *K* value of negative binomial distribution [17];

$$K=m^{2}/\left(S^{2}-m\right)$$

The spatial distribution pattern is aggregative, uniform and approximation of random when *K*>0, *K*<0 and *K*>8, respectively.

(3) Aggregation index (*I*) [18];

$$I=S^{2}/m-1$$

The spatial distribution pattern is aggregative, random and uniform when *I*>0, *I* = 0 and *I*<0, respectively.

(4) Cassie index (*C*_*a*_) [16];

$$C_{a}=(S^{2}-m)/m^{2}$$

The spatial distribution pattern is aggregative, random and uniform when *C*_*a*_>0, *C*_*a*_ = 0 and *C*_*a*_<0, respectively.

(5) Mean crowding (m*) [19];

$$m^{*}=m+(S^{2}/m-1)$$

The spatial distribution pattern is aggregative, random and uniform when m*>*m*, m* = *m* and m*<*m*, respectively.

(6) Patch index (m*/*m*) [19];

$$m^{*}/m=1+S^{2}/m^{2}-1/m$$

The spatial distribution pattern is aggregative, random and uniform when m*/*m*>1, m*/*m* = 1 and m*/*m*<1, respectively.

(7) Iwao regression [20];

$$m^{*}=\alpha +\beta m$$

When *α* = 0, the component of the distribution is a single individual; *α*>0, individuals are attracted to each other and the individual colony is the basic component in the distribution; *α*<0, shows mutual exclusion between individuals. The spatial distribution pattern is uniform, random and aggregative when *β*<1, *β* = l and *β*>1, respectively.

In addition, when *α* = 0 and *β* = l, the spatial distribution pattern is random; *α*<0 and *β*>1 refers to an aggregated negative binomial distribution with a common K; *α*>0 and *β* = 1, is an aggregated Neyman distribution or Poisson negative binomial distribution; *α*>0 and *β*>1, indicates an aggregated general negative binomial distribution; *α*<0 and *β*<1, is a uniform distribution.

(8) Taylor power law [21];

$$lgS^{2}=lga+blgm\;(S^{2}=am^{b})$$

when *lga* = 0 and b = 0, the spatial distribution pattern is random; when *lga* > 0 and b = 1, the distribution is an aggregation and the level of aggregation does not rely on density; when *lga* > 0 and b>1 occurs, it is also an aggregation, but the level of aggregation does relys on density; when *lga*> 0 and b<1, is uniform, means that there is a higher population density with more uniform distribution.

### Analysis of causes of aggregation

The population aggregations mean (*λ*) [22] was used to analysis the causes for the insect population being in an aggregated state, and was calculated as follows:

$$\lambda =m/2K^{*}y$$

where *y* equals to $X_{0.5}^{2}$ when the value of the degree of freedom is 2*K*. The aggregation of insect individuals is caused by environmental factors when *λ*<2; on the other hand, if *λ*>2, the phenomenon is caused by aggregation behavior or the aggregation behavior works in combination with the environment.

## Results

### Seasonal dynamics of *P*. *crisonalis*

**Fig 1:**
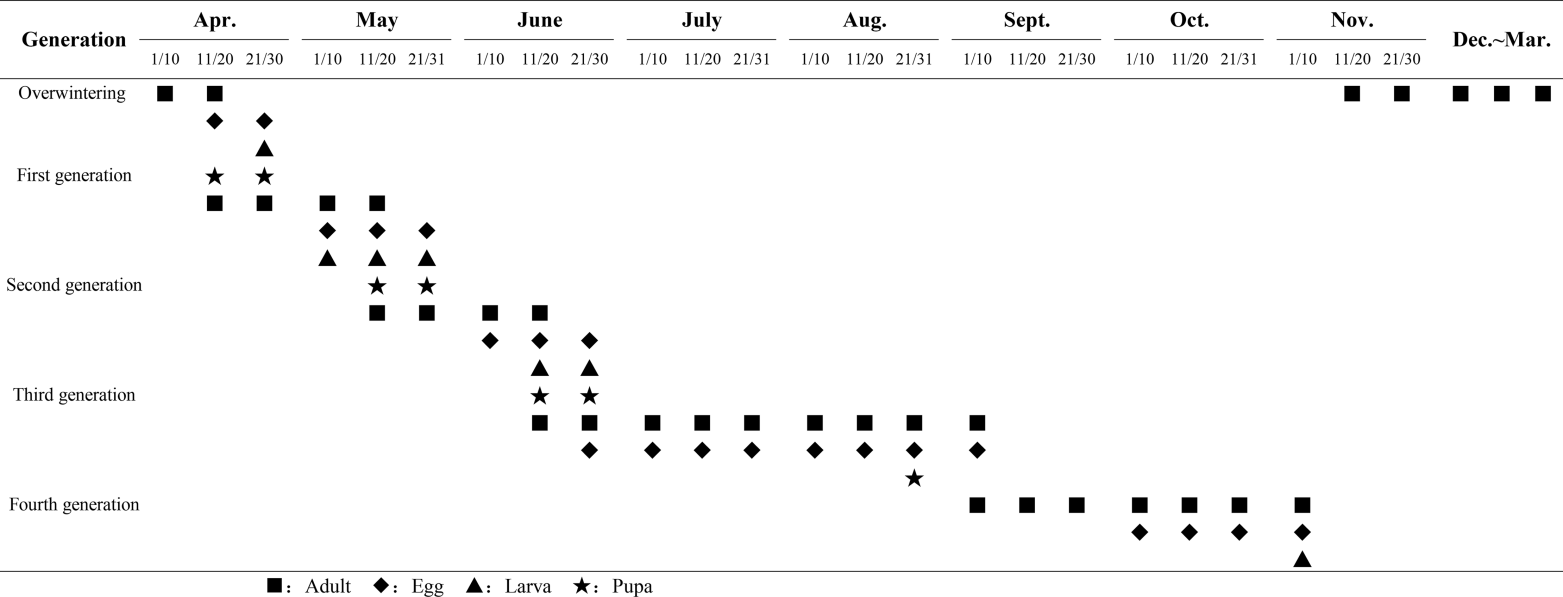
Annual life cycle data of *P*. *crisonalis* on *T*. *natans*.

**Fig 2:**
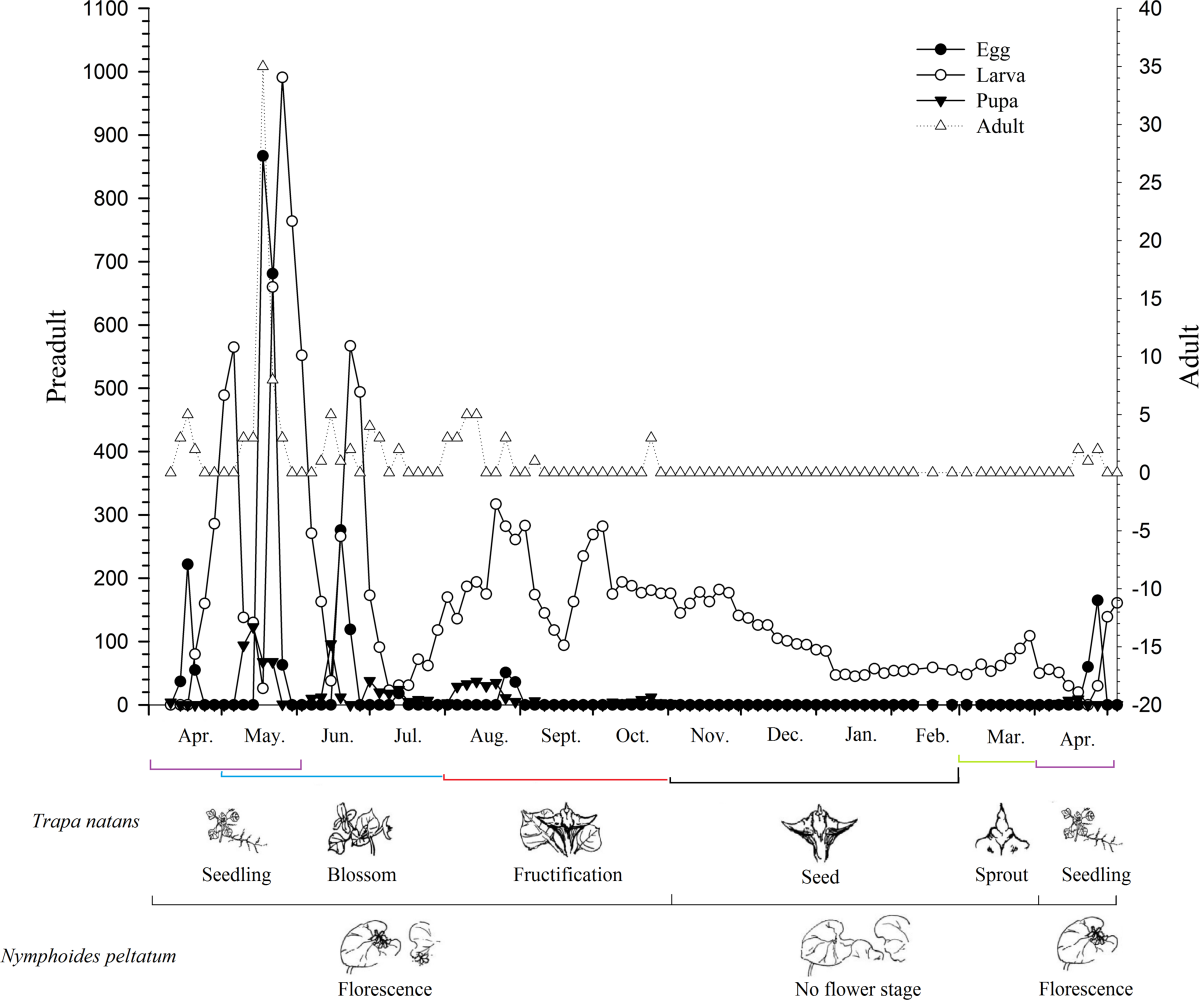
Seasonal dynamics of *P*. *crisonalis* on *T*. *natans* and *N*. *peltatum*.

**Fig 3:**
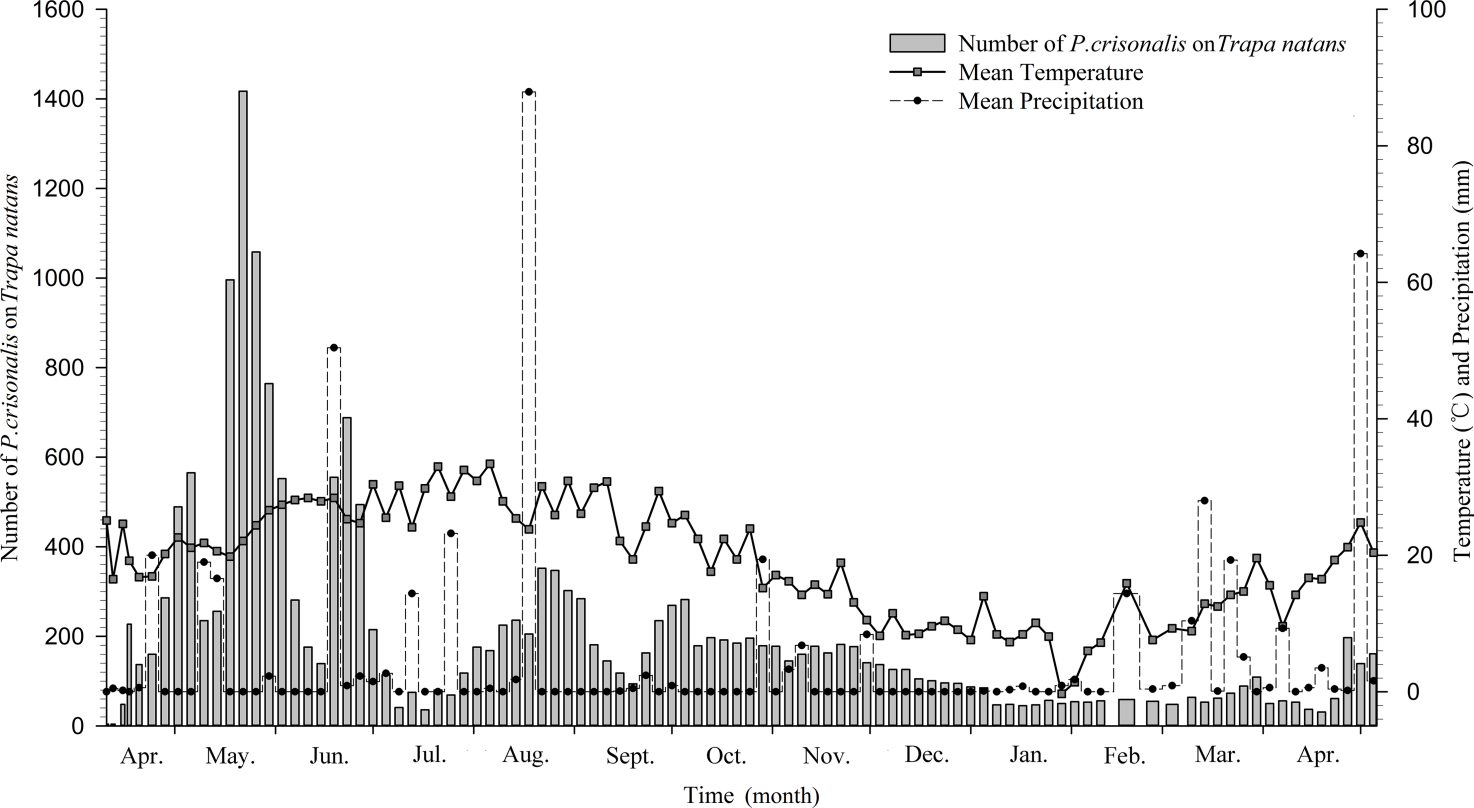
Variations of a *P*. *crisonalis* population with mean temperatures and mean precipitation.

Based on the annual life history data for *P*. *crisonalis* on *T*. *natans* (Fig 1), it is evident that *P*. *crisonalis* completed five generations per year in Changsha, and that the third generation had the longest duration and the most obvious generational overlap. The *P*. *crisonalis* population reached their peaks in mid to late May, mid-June to early July, and mid to late August to early September (Figs 2 and 3). The corresponding development stages of the major host plant (*T*. *natans*) during these time periods were seedling, blossom and fructification, while, the secondary host plant (*N*. *peltatum*) was in florescence. The mean temperatures in May, June, July, August and September for the two years was 23, 26.6, 29.7, 28.1 and 25.7°C, respectively. The *P*. *crisonalis* population decreased when periods of heavy precipitation occurred in the middle of June and August 2014 and in early May 2015.

### Spatial distribution pattern of *P*. *crisonalis*

The different aggregation indices for the four developmental stages, including the egg, larva, pupa and adult stage, are shown in Tables 1 to 4. As shown in Table 1, the diffusion coefficient (*C*) and patch index (m*/*m*) at the *P*. *crisonalis* egg stage during May and June were greater than 1.0000. The *K* value of the negative binomial distribution, aggregation index (*I*) and Cassie index (*C*_*a*_) were greater than 0.0000. The mean crowding (m*) was greater than the mean density (*m*). These indices, however, had changed in July and August when the diffusion coefficient (*C*) and patch index (m*/*m*) were less than 1.0000. The *K* value of the negative binomial distribution, the aggregation index (*I*) and Cassie index (*C*_*a*_) were less than 0.0000, and the mean crowding (m*) was smaller than the mean density (*m*), indicating that the *P*. *crisonalis* eggs were in an aggregated distribution in May and June and in a uniform distribution in July and August.

**Table 1:** Mean density, variance and indices of aggregation in the egg stage of *P*. *crisonalis*.

Month	*m*	*S*^2^	*C*	m*	m*/*m*	*K*	*C*_*a*_	*I*	*λ*
5	1.4953	8.2734	5.5328	6.0281	4.0313	0.3299	3.0313	4.5328	0.6731
6	0.2240	0.2334	1.0420	0.2660	1.1874	5.3363	0.1874	0.4198	0.2102
7	0.0113	0.0051	0.4499	-0.5388	-47.8951	-0.0205	-48.8951	-0.5501	-
8	0.0780	0.0568	0.7287	-0.1933	-2.4779	-0.2875	-3.4779	-0.2713	-

**Table 2:** Mean density, variance and indices of aggregation in the larva stage of *P*. *crisonalis*.

Month	*m*	*S*^2^	*C*	m*	m*/*m*	*K*	*C*_*a*_	*I*	*λ*
5	1.7387	2.4674	1.4191	2.1578	1.2411	4.1482	0.2411	0.4191	1.6005
6	1.5407	0.7385	0.4793	1.0200	0.6621	-2.9590	-0.3379	-0.5207	-
7	0.3163	0.0429	0.1356	-0.5481	-1.7331	-0.3659	-2.7331	-0.8644	-
8	0.8047	0.0344	0.0428	-0.1526	-0.1896	-0.8406	-1.1896	-0.9572	-
9	0.6693	0.0953	0.1424	-0.1882	-0.2812	-0.7805	-1.2812	-0.8576	-
10	0.8088	0.2253	0.2786	0.0873	0.1080	-1.1211	-0.8920	-0.7214	-
11	0.6679	0.0500	0.0749	-0.2573	-0.3852	-0.7219	-1.3852	-0.9251	-

**Table 3:** Mean density, variance and indices of aggregation in the pupa stage of *P*. *crisonalis*.

Month	*m*	*S*^2^	*C*	m*	m*/*m*	*K*	*C*_*a*_	*I*	*λ*
5	0.2040	0.0701	0.3437	-0.4523	-2.2171	-0.3108	-3.2171	-0.6563	-
6	0.0793	0.0251	0.3161	-0.6046	-7.6211	-0.1160	-8.6211	-0.6839	-
7	0.0731	0.0090	0.1237	-0.8032	-10.9833	-0.0834	-11.9833	-0.8763	-
8	0.1007	0.0073	0.0728	-0.8265	-8.2104	-0.1086	-9.2104	-0.9272	-
9	0.0020	9.73E-05	0.0486	-0.9494	-474.6757	-0.0021	-475.6757	-0.9514	-
10	0.0131	0.0007	0.0566	-0.9302	-70.8758	-0.0139	-71.8758	-0.9434	-

**Table 4:** Mean density, variance and indices of aggregation in the adult stage of *P*. *crisonalis*.

Month	*m*	*S*^2^	*C*	m*	m*/*m*	*K*	*C*_*a*_	*I*	*λ*
5	0.0327	0.0028	0.0858	-0.8815	-26.9850	-0.0357	-27.9850	-0.9142	-
6	0.0060	0.0003	0.0446	-0.9494	-158.2342	-0.0063	-159.2342	-0.9554	-
7	0.0062	0.0004	0.0646	-0.9292	-148.6709	-0.0067	-149.6709	-0.9354	-
8	0.0120	0.0007	0.0554	-0.9326	-77.7162	-0.0127	-78.7162	-0.9446	-
9	0.0007	3.33E-05	0.0500	-0.9493	-1424.0000	-0.0007	-1425.0000	-0.9500	-
10	0.0013	6.17E-05	0.0494	-0.9494	-759.5063	-0.0013	-760.5063	-0.9506	-

The aggregation indices for the larval stage are shown in Table 2. In May, the diffusion coefficient (*C*) and patch index (m*/*m*) were greater than 1.0000. The mean crowding (m*) was greater than the mean density (*m*). The other indices, including the *K* value of the negative binomial distribution, aggregation index (*I*), and Cassie index (*C*_*a*_) are all higher than 0.0000. This would indicate that during May, *P*. *crisonalis* larvae were in an aggregated distribution. From June to November, however, the diffusion coefficient (*C*) and patch index (m*/*m*) are less than 1.0000, the *K* value of the negative binomial distribution, the aggregation index (*I*) and Cassie index (*C*_*a*_) are all less than 0.0000, and the mean crowding (m*) is less than the mean density (*m*). These data show that the *P*. *crisonalis* larvae were in a uniform distribution from June to November.

The aggregation indices at the pupal stage from May to October are shown in Table 3, the diffusion coefficient (*C*) and patch index (m*/*m*) are less than 1.0000. The mean crowding (m*) is less than the mean density (*m*). The *K* value of the negative binomial distribution, aggregation index (*I*) and Cassie index (*C*_*a*_) are all less than 0.0000, meaning that the spatial distribution pattern of *P*. *crisonalis* pupae from May to October is uniform.

The trend of aggregation indices from May to October in the *P*. *crisonalis* adult stage was similar to the pupal stage (Table 4). The spatial distribution pattern of *P*. *crisonalis* adults in water chestnut ponds from May to October was also uniform.

**Fig 4:**
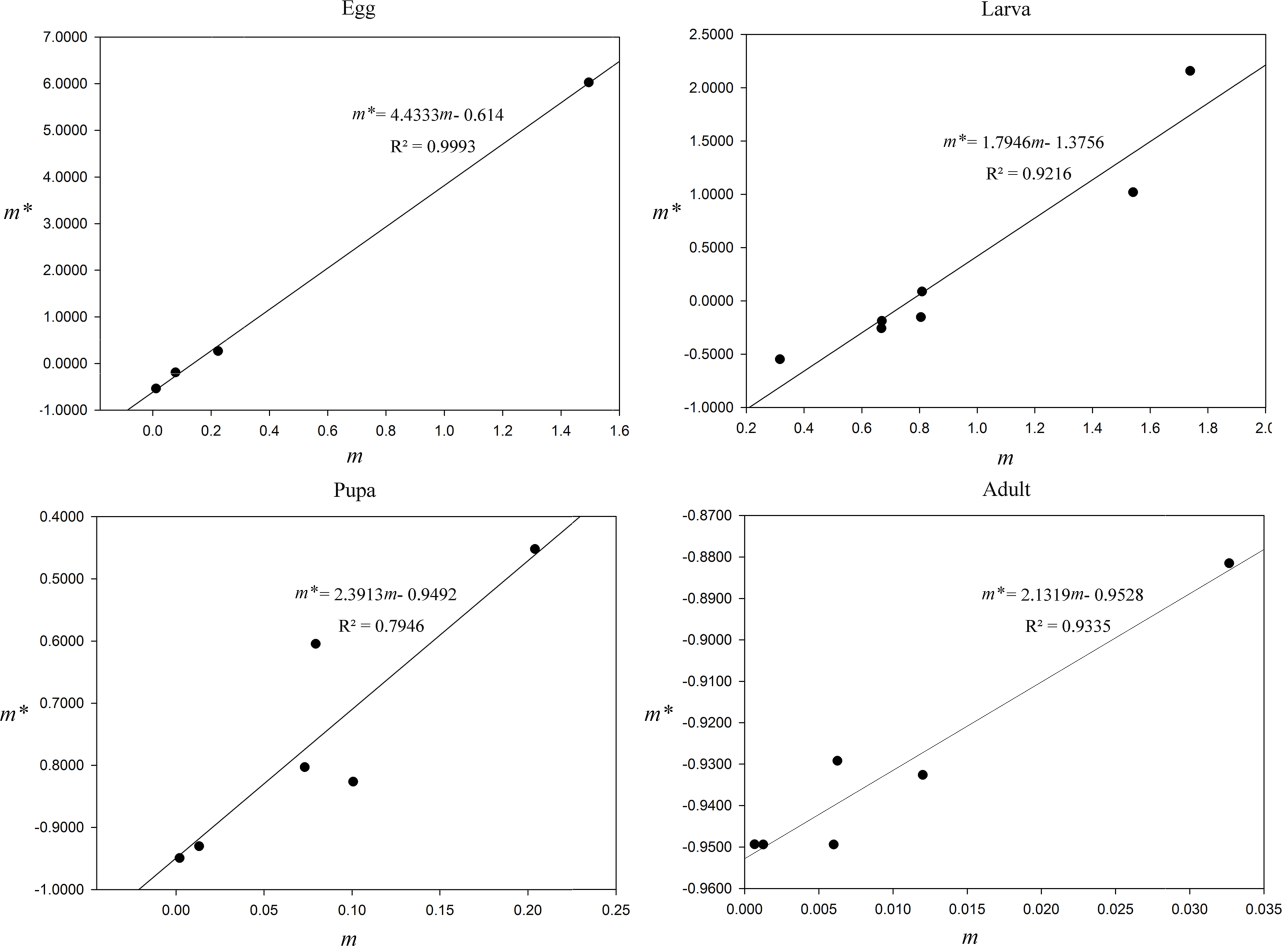
Relationship between mean crowding (m*) and mean density (*m*).

Using the Iwao regression to describe the relationship between mean crowding (m*) and mean density (*m*) during different developmental stages (Fig 4), further illustrates the distribution type of the *P*. *crisonalis* population. The regression equations of a straight line at the egg, larval, pupal and adult stages are m* = 4.4333*m*-0.614 (*r* = 0.9993), m* = 1.7946*m*-1.3756 (*r* = 0.9216), m* = 2.3913*m*-0.9492 (*r* = 0.7946) and m* = 2.1319*m*-0.9528 (*r* = 0.9335), respectively. The values of *α* in the four equations are all less than 0.0000, while all of the *β* values are more than 1.0000. These data imply that individuals are mutually exclusive in the aggregation distribution.

**Fig 5:**
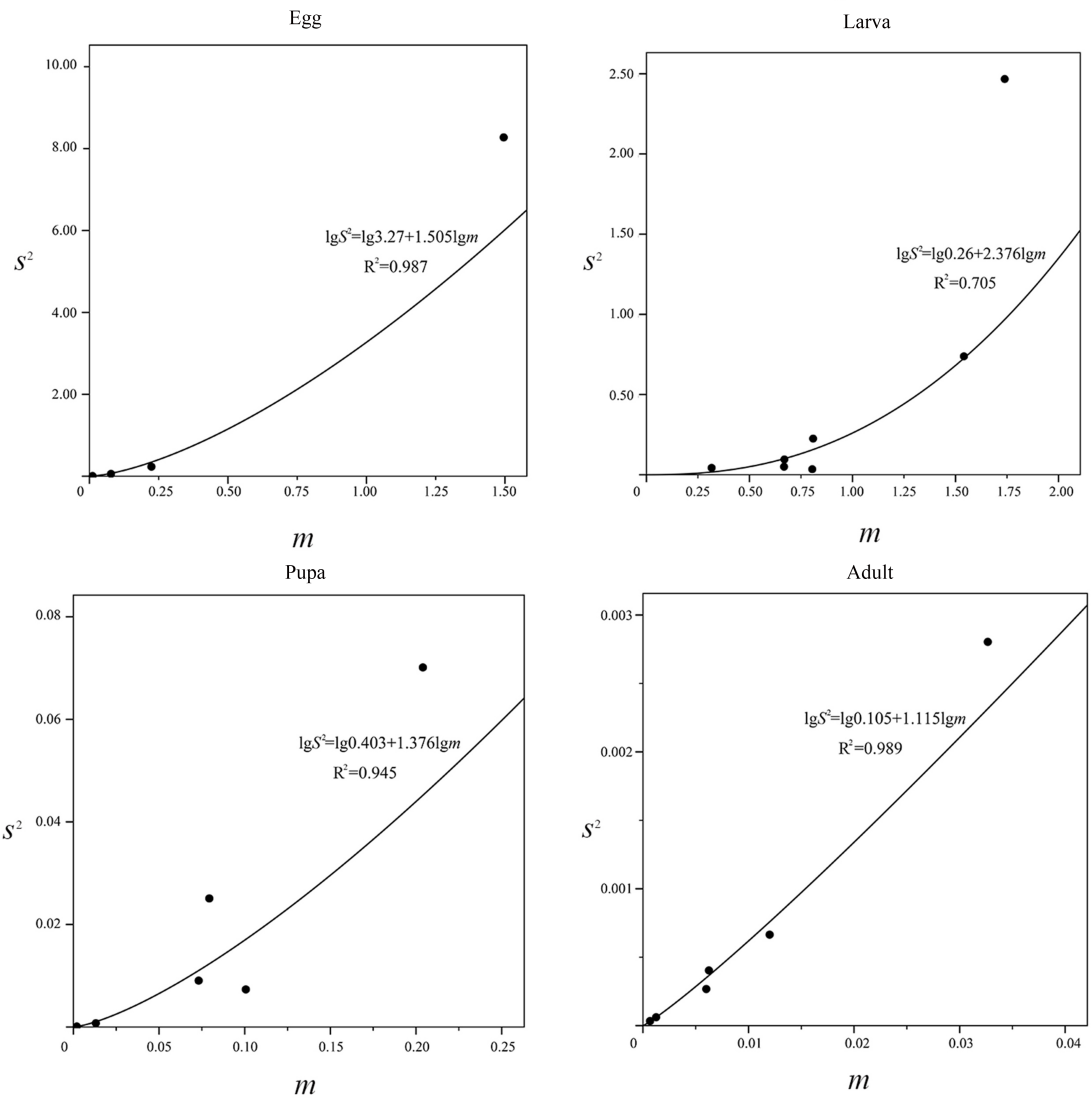
Relationship between variance (*S*^2^) and mean density (*m*).

The “Taylor power law” was used to analyze the relationship between the level of aggregation and the mean density (Fig 5). The equations of variance (*S*^2^) and mean density (*m*) at the egg, larva, pupa and adult stages were *lgS*^2^ = *lg*3.27 + 1.505*lgm*, *lgS*^2^ = *lg*0.26 + 2.376*lgm*, *lgS*^2^ = *lg*0.403 + 1.376*lgm* and *lgS*^2^ = *lg*0.105 + 1.115*lgm* respectively. Only at the egg stage, was *lga*>0 and *b*>1, that the spatial distribution at the egg stage was an aggregation and the aggregation level relies on density.

It is possible to analyze the causes of aggregation using these aggregation indices. The values of *λ* at the egg stage during May and June were 0.6731 and 0.2102, respectively; while the value at the larva stage in May was 1.6005. The *λ* values were all less than 2.0000, however, indicating that the aggregation phenomenon may be caused by environment variations.

## Discussion

The *P*. *crisonalis* population tended to increase corresponding to the seasonal changes—three population peaks appeared at the end of spring, and the beginning or end of summer, and the beginning of autumn (Fig 3). This phenomenon reflected the climatic conditions and the growth status of the host plants in Changsha. Population fluctuations and peak activities are more evident during favorable climatic conditions [23, 24]. The *P*. *crisonalis* population increased gradually when suitable temperatures occurred. In April, the mean temperature (19.4°C) was too cool for significant host plants growth to occur. Since the majority of host plants were still quite small and only beginning spring growth at this time, they would not be able to provide adequate nutrition to sustain a large increase in *P*. *crisonalis* growth. Consequently, *P*. *crisonalis* population was relatively small in April. However, by the beginning of May, the *P*. *crisonalis* population began to change, which was likely due to the increased temperature and precipitation. The *P*. *crisonalis* population was significantly reduced as a result of the heavier than normal rainfall, but rapidly increased afterwards. On the one hand, a correlation study revealed that rainfall has a significant adverse impact on the insects’ population [24, 25, 26], while, on the other hand, the wet conditions caused by the increased rainfall with the accompanying increase in environment humidity, contributed to increases in egg development and adult growth [27, 28]. By May and June, the mean temperature was 23.0°C and 26.6°C, respectively. This temperature range was approaching the insects’ optimal developmental temperature range of 24~30°C. In addition, the host plants were completing their initial stage of development (Fig 2), meaning that the plants’ maturity increased with the rising temperature and increase in precipitation. The ideal conditions present at this time all contributed to development of the *P*. *crisonalis* population and allowed them to gradually reach their annual peak. However, in late June, the mean temperature reached 29.7°C with the highest temperature over 35°C as a result of the “Northwest Pacific subtropical high” weather effects. In response to the higher temperatures the *P*. *crisonalis* population decreased rapidly and remained at a low level. In August, a period of heavy precipitation brought in lowered temperatures, allowing the *P*. *crisonalis* population to begin recovery. The high humidity, which coincided with more suitable temperatures, was conducive to a buildup of pest numbers [29]. Heavy precipitation normally causes a short-term reduction in the population, but then a rapid rebound often occurs. A warmer than average autumn caused a slight increase of the population during September, which was followed by a decreasing *P*. *crisonalis* population reflecting the decreasing fall temperatures. The *P*. *crisonalis* population continued aging until disappearing in October. In November, *P*. *crisonalis* are found overwintered in weeds, dead leaves or in soil. Although temperature plays a critical role in *P*. *crisonalis* seasonal dynamics, the effects of precipitation on the population can also be substantial.

In addition to the effects of climate, host plant selection may also play a role in the establishment of the *P*. *crisonalis* population. *P*. *crisonalis* larvae usually prefer to hide between two host plant leaves while feeding on the surface of the leaves [8]. Water chestnut (*T*. *natans*), which is the primary host of *P*. *crisonalis*, is in the seed or bud stage from November to March. Beginning in April, the host plant leaves begin to unfold providing food resources for *P*. *crisonalis*. Apparently, the *P*. *crisonalis* population begins to increase and peak after that time. The host plants provide nutrition enabling the establishment of *P*. *crisonalis* populations. Conversely, previous research has also found that quality of the host plants can affect the fecundity of herbivorous insects not only at the individual level, but also, on the population level as well [30]. It has been shown that host plants and herbivorous insects often affect each other. In addition to climate conditions and host plants effects, other pests found on water chestnut, such as *Galerucella birmanica* may also impact the *P*. *crisonalis* population [31].

Many factors can cause an insect population to exist in different spatial patterns. According to our monthly aggregation indices, the majority of *P*. *crisonalis* individuals at various development stages are in a uniform distribution. This may be a reflection of the stable water chestnut pond system, the relatively light use of pesticides and fertilizers and lack of human interference. However, *P*. *crisonalis* eggs were found to be an aggregated distribution during May and June and larvae were found in a similar distribution in May. In May and June, the host plants, *T*. *natans*, are beginning to produce seeds and are in the early blossom stage. The limited resources available may be cause of the aggregation. A similar conclusion was found to occur in distribution of eggs in the lycaenid, *Virachola livia* (Klug) [32]. Additionally, all the *λ* values were less than 2 indicating that environment variations may be the cause of the aggregation phenomenon. The Iwao m*−*m* results showed that individuals in the egg stage are mutually exclusive in the aggregation distribution; while, concurrently the Taylor power law showed the spatial distribution at the egg stage was aggregation. Aggregation levelS relys on density. A higher mean density may lead to intraspecific competition.

In Changsha, *P*. *crisonalis* normally produces five generations a year, with the third generation being the longest and having the most obvious generational overlap. This generation is responsible for causing the most serious damage, and usually coincides with the high humidity and more suitable temperatures found during this time period. Based on this information, initial control measures for *P*. *crisonalis* should be undertaken in April. The period from May to July is critical for control. Using integrated control, including traps, natural enemies, chemical pesticides and other measures should effectively control *P*. *crisonalis* populations.

## Supporting information

S1 Data Set Fig 2 Population dynamics of *Parapoynx crisonalis* at *Trapa natans* and *Nymphoides peltatum*. (DOCX)

S2 Data Set Fig 3 Variations of a *Parapoynx crisonalis* population with mean temperatures and mean precipitation. (DOCX)

S3 Data Set Fig 4 Relationship between mean crowding (m*) and mean density (*m*). (DOCX)

S4 Data Set Fig 5 Relationship between variance (*S*^2^) and mean density (*m*). (DOCX)

## References

[1] Regier JC, Mitter C, Solis M, Hayden JE, Landry B, Nuss M, et al A molecular phylogeny for the pyraloid moths (Lepidoptera: Pyraloidea) and its implications for higher level classification. Systematic Entomology, 2012; 37 (4): 635–656.

[2] Chen F, Song S, Wu C. A review of the genus *Parapoynx* Hübner in China (Lepidoptera: Pyralidae: Acentropinae). Aquatic Insect, 2006; 28 (4): 291–303.

[3] Huang GH, Li JH (eds.). Color Handbook of Insect Pests of Aquatic Vegetables in China. Wuhan: Hubei Science and Technology Press, 2013 pp. 109–111.

[4] Davis AM. A Review of the Status of Microlepidoptera in Britain. England: Butterfly Conservation Report No. 2012; S12-02.

[5] Chen Q, Chen ZS, Gu XS, Ma L, Wang X, Huang GH. The complete mitogenome of *Parapoynx crisonalis* (Walker, 1859) (Lepidoptera: Crambidae), with phylogenetic relationships among three acentropine larval forms. Aquatic Insect, 2017; 38 (1–2): 79–91.

[6] Yen SH. Insecta: Lepidoptera, Crambidae, Acentropinae. Freshwater Invertebrates of the Malaysian Region, 2004; 545–554.

[7] You P. Aquatic Lepidoptera: Nymphulinae (Pyralidae). Entomological Knowledge, 2005; 42: 595–598.

[8] Bennett CA, Buckingham GR. The herbivorous insect fauna of a submersed weed, Hydrilla verticillata (Alismatales: Hydrocharitaceae). Proceedings of the X International Symposium on Biological Control of Weeds. Montana State University, Bozeman, Montana, USA, 1999; 307–313.

[9] Chen Q, Li N, Wang X, Ma L, Huang JB, Huang GH. Age-stage, two-sex life table of *Parapoynx crisonalis* (Lepidoptera: Pyralidae) at different temperatures. PLoS ONE, 2017; 12 (3): e0173380 doi: 10.1371/journal.pone.0173380 28264022 10.1371/journal.pone.0173380 PMC5338836

[10] Orsted M, Schou MF, Kristensen TN. Biotic and abiotic factors investigated in two Drosophila species—evidence of both negative and positive effects of interactions on performance. Scientific Reports, 2017; 7: 40132 doi: 10.1038/srep40132 28059144 10.1038/srep40132 PMC5216344

[11] Mironidis GK. Development, survivorship and reproduction of *Helicoverpa armigera* (Lepidoptera: Noctuidae) under fluctuating temperatures. Bulletin of Entomological Research, 2014; 104 (6): 751–64 doi: 10.1017/S0007485314000595 25208831 10.1017/S0007485314000595

[12] Sala OE, Chapin FS, Armesto JJ, Berlow E, Bloomfield J, Dirzo R, et al Global biodiversity scenarios for the year 2100. Science, 2000; 287 (5459): 1770–1774. 10710299 10.1126/science.287.5459.1770

[13] Jumean Z, Wood C, Gries G. Frequency distribution of larval codling moth, *Cydia pomonella* L., aggregations on trees in unmanaged apple orchards of the Pacific Northwest. Environmental Entomology, 2009; 38 (5): 1395–1399. 19825294 10.1603/022.038.0507

[14] Sánchez NE, Pereyra PC, Luna MG. Spatial patterns of parasitism of the solitary parasitoid *Pseudapanteles dignus* (Hymenoptera: Braconidae) on *Tuta absoluta* (Lepidoptera: Gelechiidae). Environmental Entomology, 2009; 38 (2): 365–374. 19389284 10.1603/022.038.0208

[15] Pena JE, Schaffer B. Intraplant distribution and sampling of the *Citrus leafminer* (Lepidoptera: Gracillariidae) on lime. Journal of Economic Entomology, 1997; 90 (2): 458–464. 10.1603/0022-0493-93.2.374 10826188

[16] Cassie RM. Frequency distribution models in the ecology of plankton and other organisms. Journal of Animal Ecology, 1962; 31 (1): 65–92.

[17] Waters WE. A quantitative measure of aggregation in insects. Journal of Economic Entomology, 1959; 52 (6): 1180–1184.

[18] David FN, Moore PG. Notes on contagious distributions in plant populations. Annals of Botany, 1954; 18 (69): 47–53.

[19] Lloyd M. Mean crowding. Journal of Animal Ecology, 1967; 36 (1):1–30.

[20] Iwao S. A new regression method for analyzing the aggregation pattern of animal populations. Researches on Population Ecology, 1968; 10 (1): 1–20.

[21] Taylor LR. Aggregation, variance and the mean. Nature, 1961; 189 (4766): 732–735.

[22] Blackith RE. The water reserves of hatchling locusts. Comparative Biochemistry & Physiology, 1961; 3 (2): 99–107.

[23] Sedaratian A, Fathipour Y, Talebi AA, Farahani S. Population density and spatial distribution pattern of *Thrips tabaci* (Thysanoptera: Thripidae) on different soybean varieties. Journal of Agricultural Science and Technology, 2010; 12 (3): 275–288.

[24] Li Z, Zalucki MP, Yonow T, Kriticos DJ, Bao H, Chen H, et al Population dynamics and management of diamondback moth (*Plutella xylostella*) in China: the relative contributions of climate, natural enemies and cropping patterns. Bulletin of Entomological Research, 2016; 106 (2): 197–214. doi: 10.1017/S0007485315001017 26693884 10.1017/S0007485315001017

[25] Kuussaari M, Rytteri S, Heikkinen RK, Heliola J, von Bagh P. Weather explains high annual variation in butterfly dispersal. Proceedings of The Royal Society B-Biological Sciences. 2016; 283 (1835): 20160413. 10.1098/rspb.2016.0413 PMC4971196 27440662

[26] Rosser N, Dasmahapatra KK, Mallet J. Stable *Heliconius* butterfly hybrid zones are correlated with a local rainfall peak at the edge of the Amazon basin. Evolution, 2014; 68(12): 3470–3484. doi: 10.1111/evo.12539 25311415 10.1111/evo.12539

[27] Tamburini G, Marini L, Hellrigl K, Salvadori C, Battisti A. Effects of climate and density-dependent factors on population dynamics of the pine processionary moth in the Southern Alps. Climatic Change, 2013; 121 (4): 701–712.

[28] Lu YH, Wu KM. Effect of relative humidity on population growth of *Apolygus lucorum* (Heteroptera: Miridae). Applied Entomology and Zoology, 2011; 46 (3): 421–427.

[29] Zainab S, Ram B, Singh RN. Environmental effect on yellow stem borer, *Scirpophaga incertulas* (Walker) and rice leaf folder, *Cnaphalocrocis medinalis* (Guenee) on rice crop. Journal of Environmental Biology, 2017; 38 (2): 291–295.

[30] Awmack CS, Leather SR. Host plant quality and fecundity in herbivorous insects. Annual Review of Entomology, 2002; 47: 817–844. doi: 10.1146/annurev.ento.47.091201.145300 11729092 10.1146/annurev.ento.47.091201.145300

[31] Ding J, Wang Y, Jin X. Monitoring populations of *Galerucella birmanica* (Coleoptera: Chrysomelidae) on *Brasenia schreberi* and *Trapa natans* (Lythraceae): Implications for biological control. Biological Control, 2007; 43 (1): 71–77.

[32] Mokhtar AM, Nabhani SSA. Distribution of *Virachola livia* (Lepidoptera: Lycaenidae) eggs and influence of conspecific aggregation and avoidance behavior. Journal of Agricultural Science and Technology, 2016; 18 (6): 1593–1604.

