# Supplementary material for: Seasonal dynamics and spatial distribution pattern of Parapoynx crisonalis (Lepidoptera: Crambidae) on water chestnuts
Source: PLoS One. 2017 Sep 1;12(9):e0184149. doi: 10.1371/journal.pone.0184149 (PMC5581192; doi:10.1371/journal.pone.0184149)
Supplement: S1 Data Set — (DOCX) [file pone.0184149.s001.docx]

**S1 Data Set. Fig 2 Population dynamics of *Parapoynx crisonalis* at *Trapa natans* and *Nymphoides peltatum*.**

| Stage  Time | Egg | Larva | Pupa | Adult |
| --- | --- | --- | --- | --- |
| 10/4/2014 | 0 | 0 | 4 | 0 |
| 14/4/2014 | 37 | 0 | 0 | 3 |
| 17/4/2014 | 222 | 0 | 0 | 5 |
| 20/4/2014 | 55 | 80 | 0 | 2 |
| 24/4/2014 | 0 | 160 | 0 | 0 |
| 28/4/2014 | 0 | 286 | 0 | 0 |
| 2/5/2014 | 0 | 489 | 0 | 0 |
| 6/5/2014 | 0 | 565 | 0 | 0 |
| 10/5/2014 | 0 | 138 | 94 | 3 |
| 14/5/2014 | 0 | 130 | 123 | 3 |
| 18/5/2014 | 867 | 26 | 68 | 35 |
| 22/5/2014 | 681 | 660 | 68 | 8 |
| 26/5/2014 | 63 | 991 | 1 | 3 |
| 30/5/2014 | 0 | 764 | 0 | 0 |
| 3/6/2014 | 0 | 552 | 0 | 0 |
| 7/6/2014 | 0 | 271 | 10 | 0 |
| 11/6/2014 | 0 | 163 | 12 | 1 |
| 15/6/2014 | 0 | 38 | 96 | 5 |
| 19/6/2014 | 276 | 266 | 12 | 1 |
| 23/6/2014 | 119 | 567 | 0 | 2 |
| 27/6/2014 | 0 | 494 | 0 | 0 |
| 1/7/2014 | 0 | 173 | 38 | 4 |
| 5/7/2014 | 0 | 91 | 20 | 3 |
| 9/7/2014 | 0 | 23 | 18 | 0 |
| 13/7/2014 | 18 | 31 | 24 | 2 |
| 17/7/2014 | 0 | 31 | 5 | 0 |
| 21/7/2014 | 0 | 72 | 8 | 0 |
| 25/7/2014 | 0 | 62 | 7 | 0 |
| 29/7/2014 | 0 | 118 | 0 | 0 |
| 2/8/2014 | 0 | 170 | 3 | 3 |
| 6/8/2014 | 0 | 136 | 29 | 3 |
| 10/8/2014 | 0 | 187 | 33 | 5 |
| 14/8/2014 | 0 | 194 | 37 | 5 |
| 18/8/2014 | 0 | 175 | 30 | 0 |
| 22/8/2014 | 0 | 317 | 35 | 0 |
| 26/8/2014 | 51 | 282 | 11 | 3 |
| 30/8/2014 | 36 | 261 | 5 | 0 |
| 3/9/2014 | 0 | 283 | 1 | 0 |
| 7/9/2014 | 0 | 174 | 6 | 1 |
| 11/9/2014 | 0 | 145 | 0 | 0 |
| 15/9/2014 | 0 | 118 | 0 | 0 |
| 19/9/2014 | 0 | 94 | 0 | 0 |
| 23/9/2014 | 0 | 163 | 0 | 0 |
| 27/9/2014 | 0 | 235 | 0 | 0 |
| 1/10/2014 | 0 | 269 | 0 | 0 |
| 5/10/2014 | 0 | 282 | 0 | 0 |
| 9/10/2014 | 0 | 175 | 4 | 0 |
| 13/10/2014 | 0 | 194 | 3 | 0 |
| 17/10/2014 | 0 | 188 | 4 | 0 |
| 21/10/2014 | 0 | 177 | 8 | 0 |
| 25/10/2014 | 0 | 181 | 12 | 3 |
| 29/10/2014 | 0 | 176 | 3 | 0 |
| 2/11/2014 | 0 | 176 | 2 | 0 |
| 6/11/2014 | 0 | 145 | 0 | 0 |
| 10/11/2014 | 0 | 160 | 0 | 0 |
| 14/11/2014 | 0 | 178 | 0 | 0 |
| 18/11/2014 | 0 | 163 | 0 | 0 |
| 22/11/2014 | 0 | 182 | 0 | 0 |
| 26/11/2014 | 0 | 177 | 0 | 0 |
| 30/11/2014 | 0 | 141 | 0 | 0 |
| 4/12/2014 | 0 | 137 | 0 | 0 |
| 8/12/2014 | 0 | 126 | 0 | 0 |
| 12/12/2014 | 0 | 126 | 0 | 0 |
| 16/12/2014 | 0 | 105 | 0 | 0 |
| 20/12/2014 | 0 | 101 | 0 | 0 |
| 24/12/2014 | 0 | 96 | 0 | 0 |
| 28/12/2014 | 0 | 95 | 0 | 0 |
| 1/1/2015 | 0 | 87 | 0 | 0 |
| 5/1/2015 | 0 | 85 | 0 | 0 |
| 9/1/2015 | 0 | 47 | 0 | 0 |
| 13/1/2015 | 0 | 48 | 0 | 0 |
| 17/1/2015 | 0 | 45 | 0 | 0 |
| 21/1/2015 | 0 | 47 | 0 | 0 |
| 25/1/2015 | 0 | 57 | 0 | 0 |
| 29/1/2015 | 0 | 50 | 0 | 0 |
| 2/2/2015 | 0 | 54 | 0 | 0 |
| 6/2/2015 | 0 | 53 | 0 | 0 |
| 10/2/2015 | 0 | 56 | 0 | 0 |
| 18/2/2015 | 0 | 59 | 0 | 0 |
| 26/2/2015 | 0 | 55 | 0 | 0 |
| 4/3/2015 | 0 | 48 | 0 | 0 |
| 10/3/2015 | 0 | 64 | 0 | 0 |
| 14/3/2015 | 0 | 53 | 0 | 0 |
| 18/3/2015 | 0 | 62 | 0 | 0 |
| 22/3/2015 | 0 | 73 | 0 | 0 |
| 26/3/2015 | 0 | 89 | 0 | 0 |
| 30/3/2015 | 0 | 109 | 0 | 0 |
| 3/4/2015 | 0 | 50 | 0 | 0 |
| 7/4/2015 | 0 | 56 | 0 | 0 |
| 11/4/2015 | 0 | 51 | 2 | 0 |
| 15/4/2015 | 0 | 30 | 7 | 0 |
| 19/4/2015 | 0 | 20 | 9 | 2 |
| 23/4/2015 | 60 | 0 | 0 | 1 |
| 27/4/2015 | 165 | 30 | 0 | 2 |
| 1/5/2015 | 0 | 139 | 0 | 0 |
| 5/5/2015 | 0 | 161 | 0 | 0 |
