# Supplementary material for: Seasonal dynamics and spatial distribution pattern of Parapoynx crisonalis (Lepidoptera: Crambidae) on water chestnuts
Source: PLoS One. 2017 Sep 1;12(9):e0184149. doi: 10.1371/journal.pone.0184149 (PMC5581192; doi:10.1371/journal.pone.0184149)
Supplement: S2 Data Set — (DOCX) [file pone.0184149.s002.docx]

**S2 Data Set. Fig 3 Variations of a *Parapoynx crisonalis* population with temperatures and precipitation.**

| Time | Mean temperature | Mean precipitation | Number of *P.crisonalis* |
| --- | --- | --- | --- |
| 15/4/2014 | 24.6 | 0.2 | 48 |
| 17/4/2014 | 19.2 | 0 | 227 |
| 20/4/2014 | 16.8 | 0.6 | 137 |
| 24/4/2014 | 16.9 | 20 | 160 |
| 28/4/2014 | 20.2 | 0 | 286 |
| 2/5/2014 | 22.6 | 0 | 489 |
| 6/5/2014 | 21.1 | 0 | 565 |
| 10/5/2014 | 21.8 | 19 | 235 |
| 14/5/2014 | 20.6 | 16.6 | 256 |
| 18/5/2014 | 19.8 | 0 | 996 |
| 22/5/2014 | 22.1 | 0 | 1417 |
| 26/5/2014 | 24.4 | 0 | 1058 |
| 30/5/2014 | 26.6 | 2.3 | 764 |
| 3/6/2014 | 27.4 | 0 | 552 |
| 7/6/2014 | 28.1 | 0 | 281 |
| 11/6/2014 | 28.4 | 0 | 176 |
| 15/6/2014 | 27.9 | 0 | 139 |
| 19/6/2014 | 28.4 | 50.4 | 555 |
| 23/6/2014 | 25.3 | 0.9 | 688 |
| 27/6/2014 | 24.7 | 2.3 | 494 |
| 1/7/2014 | 30.4 | 1.5 | 215 |
| 5/7/2014 | 25.5 | 2.7 | 114 |
| 9/7/2014 | 30.2 | 0 | 41 |
| 13/7/2014 | 24.1 | 14.4 | 75 |
| 17/7/2014 | 29.8 | 0 | 36 |
| 21/7/2014 | 33 | 0 | 80 |
| 25/7/2014 | 28.6 | 23.2 | 69 |
| 29/7/2014 | 32.5 | 0 | 118 |
| 2/8/2014 | 30.9 | 0 | 176 |
| 6/8/2014 | 33.4 | 0.5 | 168 |
| 10/8/2014 | 27.9 | 0 | 225 |
| 14/8/2014 | 25.4 | 1.8 | 236 |
| 18/8/2014 | 23.8 | 87.9 | 205 |
| 22/8/2014 | 30.1 | 0 | 352 |
| 26/8/2014 | 25.9 | 0 | 347 |
| 30/8/2014 | 30.9 | 0 | 302 |
| 3/9/2014 | 26.1 | 0 | 284 |
| 7/9/2014 | 29.9 | 0 | 181 |
| 11/9/2014 | 30.8 | 0 | 145 |
| 15/9/2014 | 22.1 | 0.1 | 118 |
| 19/9/2014 | 19.4 | 0.5 | 94 |
| 23/9/2014 | 24.2 | 2.4 | 163 |
| 27/9/2014 | 29.4 | 0 | 235 |
| 1/10/2014 | 24.7 | 0.9 | 269 |
| 5/10/2014 | 25.9 | 0 | 282 |
| 9/10/2014 | 22.4 | 0 | 179 |
| 13/10/2014 | 17.6 | 0 | 197 |
| 17/10/2014 | 22.4 | 0 | 192 |
| 21/10/2014 | 19.4 | 0 | 185 |
| 25/10/2014 | 23.9 | 0 | 196 |
| 29/10/2014 | 15.2 | 19.4 | 179 |
| 2/11/2014 | 17.1 | 0 | 178 |
| 6/11/2014 | 16.2 | 3.3 | 145 |
| 10/11/2014 | 14.2 | 6.8 | 160 |
| 14/11/2014 | 15.7 | 0 | 178 |
| 18/11/2014 | 14.3 | 0 | 163 |
| 22/11/2014 | 18.9 | 0 | 182 |
| 26/11/2014 | 13.1 | 0 | 177 |
| 30/11/2014 | 10.5 | 8.4 | 141 |
| 4/12/2014 | 8.2 | 0 | 137 |
| 8/12/2014 | 11.5 | 0 | 126 |
| 12/12/2014 | 8.3 | 0 | 126 |
| 16/12/2014 | 8.5 | 0 | 105 |
| 20/12/2014 | 9.6 | 0 | 101 |
| 24/12/2014 | 10.4 | 0 | 96 |
| 28/12/2014 | 9.1 | 0 | 95 |
| 1/1/2015 | 7.6 | 0 | 87 |
| 5/1/2015 | 14 | 0.1 | 85 |
| 9/1/2015 | 8.4 | 0 | 47 |
| 13/1/2015 | 7.3 | 0.3 | 48 |
| 17/1/2015 | 8.4 | 0.8 | 45 |
| 21/1/2015 | 10.1 | 0 | 47 |
| 25/1/2015 | 8.1 | 0 | 57 |
| 29/1/2015 | -0.3 | 0.9 | 50 |
| 2/2/2015 | 1.4 | 1.8 | 54 |
| 6/2/2015 | 6 | 0 | 53 |
| 10/2/2015 | 7.2 | 0 | 56 |
| 18/2/2015 | 15.9 | 14.4 | 59 |
| 26/2/2015 | 7.6 | 0.4 | 55 |
| 4/3/2015 | 9.3 | 0.9 | 48 |
| 10/3/2015 | 8.9 | 10.4 | 64 |
| 14/3/2015 | 12.9 | 28 | 53 |
| 18/3/2015 | 12.5 | 0.1 | 62 |
| 22/3/2015 | 14.2 | 19.3 | 73 |
| 26/3/2015 | 14.7 | 5.1 | 89 |
| 30/3/2015 | 19.6 | 0 | 109 |
| 3/4/2015 | 15.6 | 0.6 | 50 |
| 7/4/2015 | 9.6 | 9.3 | 56 |
| 11/4/2015 | 14.2 | 0 | 53 |
| 15/4/2015 | 16.7 | 0.6 | 37 |
| 19/4/2015 | 16.5 | 3.5 | 31 |
| 23/4/2015 | 19.3 | 0.4 | 61 |
| 27/4/2015 | 21.2 | 0.2 | 197 |
| 1/5/2015 | 24.8 | 64.2 | 139 |
| 5/5/2015 | 20.4 | 1.6 | 161 |
