# Supplementary material for: Seasonal dynamics and spatial distribution pattern of Parapoynx crisonalis (Lepidoptera: Crambidae) on water chestnuts
Source: PLoS One. 2017 Sep 1;12(9):e0184149. doi: 10.1371/journal.pone.0184149 (PMC5581192; doi:10.1371/journal.pone.0184149)
Supplement: S3 Data Set — (DOCX) [file pone.0184149.s003.docx]

**S3 Data Set. Fig 4 Relationship between mean crowding (*m**) and mean density (*m*)**

**Egg**

| *m* | 1.495333 | 0.224 | 0.01125 | 0.078 | 0 | 0 | 0 |
| --- | --- | --- | --- | --- | --- | --- | --- |
| *m** | 6.0281 | 0.2660 | -0.5388 | -0.1933 | 0 | 0 | 0 |

**Larva**

| *m* | 1.738667 | 1.540667 | 0.31625 | 0.804667 | 0.669333 | 0.80875 | 0.667857 |
| --- | --- | --- | --- | --- | --- | --- | --- |
| *m** | 2.1578 | 1.0200 | -0.5481 | -0.1526 | -0.1882 | 0.0873 | -0.2573 |

**Pupa**

| *m* | 0.204 | 0.079333 | 0.073125 | 0.100667 | 0.002 | 0.013125 | 0 |
| --- | --- | --- | --- | --- | --- | --- | --- |
| *m** | -0.4523 | -0.6046 | -0.8032 | -0.8265 | -0.9494 | -0.9302 | 0 |

**Adult**

| *m* | 0.032667 | 0.006 | 0.00625 | 0.012 | 0.000667 | 0.00125 | 0 |
| --- | --- | --- | --- | --- | --- | --- | --- |
| *m** | -0.8815 | -0.9494 | -0.9292 | -0.9326 | -0.9493 | -0.9494 | 0 |
