# Supplementary material for: Seasonal dynamics and spatial distribution pattern of Parapoynx crisonalis (Lepidoptera: Crambidae) on water chestnuts
Source: PLoS One. 2017 Sep 1;12(9):e0184149. doi: 10.1371/journal.pone.0184149 (PMC5581192; doi:10.1371/journal.pone.0184149)
Supplement: S4 Data Set — (DOCX) [file pone.0184149.s004.docx]

**S4 Data Set. Fig 5 Relationship between variance (*S^2^*) and mean density (*m*)**

**Egg**

| *S^2^* | 8.27339009 | 0.233402703 | 0.005061709 | 0.056840541 |
| --- | --- | --- | --- | --- |
| *m* | 1.495333333 | 0.224 | 0.01125 | 0.078 |

**Larva**

| *S^2^* | 2.467403604 | 0.738492793 | 0.042897152 | 0.034403604 | 0.095330631 | 0.225302215 | 0.050002588 |
| --- | --- | --- | --- | --- | --- | --- | --- |
| *m* | 1.738666667 | 1.540666667 | 0.31625 | 0.804666667 | 0.669333333 | 0.80875 | 0.667857143 |

**Pupa**

| *S^2^* | 0.070118919 | 0.025073874 | 0.009047073 | 0.007330631 | 9.72973E-05 | 0.000743275 |
| --- | --- | --- | --- | --- | --- | --- |
| *m* | 0.204 | 0.079333333 | 0.073125 | 0.100666667 | 0.002 | 0.013125 |

**Adult**

| *S^2^* | 0.002803604 | 0.000267568 | 0.000403481 | 0.000664865 | 3.33333E-05 | 6.17089E-05 |
| --- | --- | --- | --- | --- | --- | --- |
| *m* | 0.032666667 | 0.006 | 0.00625 | 0.012 | 0.000666667 | 0.00125 |
